# Supplementary material for: Mammalian genes induce partially reprogrammed pluripotent stem cells in non-mammalian vertebrate and invertebrate species
Source: eLife. 2013 Sep 3;2:e00036. doi: 10.7554/eLife.00036 (PMC3762186; doi:10.7554/eLife.00036)
Supplement: Supplementary file 1. — (A) Controls and experimental groups. (B) Conditions used for cell derivation and maintenance. (C) Primers used for RT-PCR to amplify and quantify expression of species-specific regions of the genes. (D) p-values for the graphs shown in the figures of this study. All comparisons show Tukey’s post hoc p-values from an ANOVA test, except for titer (Figure 2—figure supplement 2) that shows the overall ANOVA p-value. Bold, significantly different at p<0.05; #, approaches significance. iPSC* = Exogenous expression of factors; iPSCs = Endogenous expression of factors; FB = fibroblast, ESC = Embryonic stem cell, EB = Embryoid body. NA = Not applicable. (E) RNA extraction of adult tissue. The contents of the table denote the origin of the adult tissues template used to compare the different levels of gene expression versus embryonic fibroblasts and iPSC-like cells. DOI: http://dx.doi.org/10.7554/eLife.00036.021 [file elife00036s001.docx]

**Supplementary file 1A. Controls and experimental groups.**

| ***Species*** | ***Experimental group*** | ***Positive Control*** | ***Negative control 1*** | ***Negative Control 2*** | ***Negative Control 3*** |
| --- | --- | --- | --- | --- | --- |
| Mouse  (*endogenous control*) | Embryonic fibroblasts transduced with EF1a-STEMCCA lentivirus grown in stem cell media | ESC derived from 129X1/SvJ x 129S1)F1 blastocysts grown in 3i stem cell media or mouse stem cell media. | Embryonic fibroblasts transduced with EF1a-GFP lentivirus grown in stem cell media | Embryonic fibroblasts transduced with EF1a-GFP lentivirus grown in complete media | Embryonic fibroblasts grown in stem cell media |
| Chicken | same as above except avian stem cell media | ESC grown in chicken embryonic stem cell media | same as above except avian stem cell media | same as above except avian complete media | same as above except avian stem cell media |
| Quail | same as chicken | none | same as chicken | same as chicken | same as chicken |
| Zebra Finch | same as chicken | none | same as chicken | same as chicken | same as chicken |
| Zebrafish | same as above except fish stem cell media | none | same as above except fish stem cell media | same as above except fish complete media | same as above except fish stem cell media |
| Drosophila | same as above except drosophila stem cell media | none | same as above except drosophila stem cell media | same as above except drosophila complete media | same as above except drosophila stem cell media |

**Supplementary file 1B. Conditions used for cell derivation and maintenance.**

| Species | **Media Conditions** |
| --- | --- |
| Mouse | **Fibroblast complete media*:** 37^o^C,5% C02; DMEM, 10% FBS, 1%Glutamax, 1% Sodium Pyruvate, 1% non-essential amino acids, 1% Penicilin/Streptomyocin, 0.5% Gentamiacin  **Mouse stem cell media**:** KO-DMEM +15% Fetal Bovine Serum (FBS; *25% for derivation*), 1% NEAA (1mM), 1% Sodium Pyruvate (1mM), 1% nucleosides (1mM), 0.5% Gentamiacin, 1% (2mM)Glutamax,0.5% Penicillin/Streptomyocin (PS) 0. 1mM Beta-Mercaptoethanol, cytokines and growth factors: LIF (Millipore, Final concentration 1000 U/ml) |
| Chicken, Quail, Finch | **Fibroblast complete media:** Same as Mouse Fibroblast Media at 39^o^C and 10%C0_2_  **Chicken ES media:** KO-DMEM +10% FBS, 1% NEAA (1mM), 1% Sodium Pyruvate (1mM), 1% nucleosides (1mM), 0.5% Gentamiacin, 1% (2mM) Glutamax, 0.5% Penicillin/Streptomyocin (PS) 0.1mM Beta-Mercaptoethanol, cytokines and growth factors:  LIF (1000 U/ml, *2000U/ml for derivation*), hrIL-6 (1ng/ml), hrsIL-6Ra (Preprotech, ref 200-06R, 1ng/ml, rmSCF (1 ng/ml), rhIGF1 (5 ng/ml), rhbFGF (1 ng/ml).  **Modified avian stem cell media:** KO-DMEM +10% FBS (20% FBS for derivation), 1% NEAA (1mM), 1% Sodium Pyruvate (1mM), 1% nucleosides (1mM), 0.5% Gentamiacin, 1% (2mM)Glutamax,0.5% Penicillin/Streptomyocin (PS) 0. 1mM Beta-Mercaptoethanol, cytokines and growth factors: LIF (1000 U/ml, *2000U/ml for derivation*), hrIL-6 (1ng/ml), hrsIL-6Ra (Preprotech, ref 200-06R, 1ng/ml, rmSCF (1 ng/ml), rhIGF1(5 ng/ml), rhbFGF (1 ng/ml), supplemented with MEK inhibitor PD0325901 (0.5 μM), ALK5 inhibitor A-83-01 (0.5 μM), GSK3β inhibitor CHIR99021 (3 μM). |
| Zebrafish | **Zebrafish Fibroblast complete media:** 28^o^C, 50% Leibovitz's L-15 medium (ATCC 30-2008), 35% Dulbecco's Modified Eagle's medium, high glucose (GIBCO 12100), 15% F12 medium (GIBCO 21700), 0.18 g/L sodium bicarbonate, 15 mM HEPES, 10% heat-inactivated fetal bovine serum, 1%PS, 0.5% Gentamiacin  **iPSC stem cell media:** Fibroblast media supplemented with LIF (1000 U/ml), MEK inhibitor PD0325901 (0.5 μM), ALK5 inhibitor A-83-01 (0.5 μM), GSK3β inhibitor CHIR99021(3 μM)). |
| Drosophila | **S2 cell line complete media:** 24^o^C, Schneider's Drosophila Medium, 90%; heat-inactivated fetal bovine serum, 10%, 1% PS, 0.5% Gentamiacin  **iPSC stem cell media :** S2 cell media supplemented with MEK inhibitor PD0325901 (0.5 μM), ALK5 inhibitor A-83-01 (0.5 μM), GSK3β inhibitor CHIR99021(3 μM)). |

*Fibroblast complete media were used for growth of fibroblast and for the initial transduction of cells, whereas stem cell media was used to derive and maintain the iPSCs and other stem cells.

**Supplementary file 1C. Primers used for RT-PCR to amplify and quantify expression of species-specific regions of the genes.**

| **STEMCCA** | **Identification** | **Fwd Primer** | **Rev Primer** |
| --- | --- | --- | --- |
| WPRE | STEMCCA | ccacctgtcagctcctttccgggactttcgct | agcgaaagtcccggaaaggagctgacaggtgg |

| **Mouse** | **Gene Identification** | **Fwd Primer** | **Rev Primer** |
| --- | --- | --- | --- |
| Oct-4 | NM_013633.2 | CCCCATGTCCGCCCGCATAC | AGGCCCAGTCCAACCTGAGGTC |
| Sox-2 | NM_011443.3 | GAAGAACAGCCCGGACCGCGT | ATGAACGGCCGCTTCTCGGT |
| c-Myc | NM_010849.4 | ACCCGCTCAACGACAGCAGC | ACTAGGGGCTCAGGGCTGGC |
| KLF-4 | NM_207209.2 | TAGTGGCGCCCTACAGCGGT | TCGTGTGTGTTGGGCCGGTG |
| GATA-4 | NM_008092.3 | GACTCACGGAGATCGCGCCG | GCCTGCTACACACCCAGGCG |
| Brachyury | NM_009309.2 | GTGTCCAACGGGCTGGGAGC | GCAGCTGAGACCGTGTGCGT |
| Nestin | [NM_016701.3](http://www.ncbi.nlm.nih.gov/entrez/viewer.fcgi?db=nucleotide&id=50363231) | AGTGCTCCACGTCCGCTTGC | AGGTAGGCCTCCAGGCGTCG |
| Vasa-Homologue | [NM_010029.2](http://www.ncbi.nlm.nih.gov/entrez/viewer.fcgi?db=nucleotide&id=225007637) | TTCAGCTTCATCAGATATTGGCGAGTC | GCCCTTTCCTCTTCCAAAGCCACC |
| Nanog | [NM_028016.1](http://www.ncbi.nlm.nih.gov/entrez/viewer.fcgi?db=nucleotide&id=110625917) | GGCTGCCTCTCCTCGCCCTT | GTGCACACAGCTGGGCCTGA |

| **Chicken/Quail** | **Gene Identification** | **Fwd Primer** | **Rev Primer** |
| --- | --- | --- | --- |
| Oct-4 | [NM_001110178](http://www.ncbi.nlm.nih.gov/nuccore/NM_001110178) | GGGAAGATGTTCAGCCAGAC | GTCTGGTTCTGCAACCGGCG |
| Sox-2 | NM_205188.1 | GCAGAGAAAAGGGAAAAAGGA | TTTCCTAGGGAGGGGTATGAA |
| c-Myc | [NM_001030952.1](http://www.ncbi.nlm.nih.gov/entrez/viewer.fcgi?db=nucleotide&id=73661205) | CGGAGAAGCTCGCCACCTACCA | CACCGAGGGGTCGATGCAGTC |
| KLF-4 | [XM_001233583.1](http://www.ncbi.nlm.nih.gov/entrez/viewer.fcgi?db=nucleotide&id=118117779) | GGCGACGGCGGCTAATTTTCG | GGCGGCCCATGGAGAAGTCG |
| Chicken Vasa | [NM_204708.1](http://www.ncbi.nlm.nih.gov/entrez/viewer.fcgi?db=nucleotide&id=45382658) | GGCGTTCTGAGGAGCAGGCG | GGGGCTGCTTGGTCTGCTGG |
| Gata-4 homologue | [XM_420041.1](http://www.ncbi.nlm.nih.gov/entrez/viewer.fcgi?db=nucleotide&id=50745251) | AACCTGGAGCCGAAAGCGCC | CCGCGATGAGGTGGAGGGGA |
| Brachyury homolog | [NM_204940.1](http://www.ncbi.nlm.nih.gov/entrez/viewer.fcgi?db=nucleotide&id=45384399) | TATTCCCGGGGCTGGGGCTC | TGGGGTAGGGGGCAGAACGG |
| Nestin | [NM_205033.1](http://www.ncbi.nlm.nih.gov/entrez/viewer.fcgi?db=nucleotide&id=45384297) | GCAGAGCCAGAGCGCACCAA | CAGGCTCAGCCCCACTGTGC |
| Nanog | [NM_001146142.1](http://www.ncbi.nlm.nih.gov/nuccore/NM_001146142.1) | CAGCAGACCTCTCCTTGACC | TTCCTTGTCCCACTCTCACC |

| **Finch** | **Gene Identification** | **Fwd Primer** | **Rev Primer** |
| --- | --- | --- | --- |
| Oct-4 | ENSTGUG00000002412 | GGGAAGATGTTCAGCCAGAC | GTCTGGTTCTGCAACCGGCG |
| Sox-2 | ENSTGUG00000010554 | GCAGAGAAAAGGGAAAAAGGA | TTTCCTAGGGAGGGGTATGAA |
| c-Myc | [ENSTGUT00000013027](http://uswest.ensembl.org/Taeniopygia_guttata/Transcript/Sequence_cDNA?db=core;g=ENSTGUG00000012513;r=2:146517055-146519434;t=ENSTGUT00000013027) | ATCGACCCCTCGGGGGGGTT | ATTCTCCCCGCGGCTCT |
| KLF4 | [XM_002188405.1](http://www.ncbi.nlm.nih.gov/entrez/viewer.fcgi?db=nucleotide&id=224091534) | TCAAGAGGGGACGCCGGTCA | TTGCCGCAGCCCGCATAGTC |
| Vasa | XP_002187022.1 | ACCATCATGTGTGAT | TACCTGACGAATCAA |
| GATA 4 | [XM_002186806.1](http://www.ncbi.nlm.nih.gov/entrez/viewer.fcgi?db=nucleotide&id=224048926) | CGCAGCGTCCCCGGTCTATG | GGCTCCGGGCTGAGTCCAGAT |
| Brachyury homolog | [XM_002187767.1](http://www.ncbi.nlm.nih.gov/entrez/viewer.fcgi?db=nucleotide&id=224047732) | AGTAACAGCAGCATCACGCCG | CAGCCCGTTGGACACTCCGC |
| Nestin | XP_002200203.1 | AACAAGCGGCTGGAGGCGTA | TTCTTCTCGAGCTACCTGCT |
| Nanog | ENSTGUG00000013123 | GCTGGTCACGATTGTGTATGCGTT | ACTGGATGAAGCTGGGGA |

| **Zebrafish** | **Gene Identification** | **Fwd Primer** | **Rev Primer** |
| --- | --- | --- | --- |
| Pou2/Pou5 | [NM_131112.1](http://www.ncbi.nlm.nih.gov/entrez/viewer.fcgi?db=nucleotide&id=18859248) | GAGCGCAGAGCCCAACAGCA | CTGCAAGGACGCTCCGCCAA |
| Sox-2 | [NM_213118.1](http://www.ncbi.nlm.nih.gov/entrez/viewer.fcgi?db=nucleotide&id=54261782) | GCCAAACGCCTTCGGGCTCT | CGCCCGGCAGGGTGTACTTG |
| MycA | [NM_131412.1](http://www.ncbi.nlm.nih.gov/entrez/viewer.fcgi?db=nucleotide&id=110626154) | TGTGGAGCGGCTTTTCCGCC | TCAGCGTGCAAAGACGCCAGT |
| KLF-4a | [NM_001113483.1](http://www.ncbi.nlm.nih.gov/entrez/viewer.fcgi?db=nucleotide&id=164698463) | CCGCGGTCCCGTTTGCATGT | CAGATCCATGGGCCGGCTGG |
| Vasa homologue | [NM_131057.1](http://www.ncbi.nlm.nih.gov/entrez/viewer.fcgi?db=nucleotide&id=18859540) | GCCTTGGCCACTGTCCGGTT | CCGGTGTTCCCACAGCGTCC |
| Gata-4 homologue | [NM_131236.1](http://www.ncbi.nlm.nih.gov/entrez/viewer.fcgi?db=nucleotide&id=18858734) | GCCTCTTGGACGGCTTCGCA | ACACTCCCGCCCTCGCAGAT |
| Brachyury homolog | [XM_001343597.2](http://www.ncbi.nlm.nih.gov/entrez/viewer.fcgi?db=nucleotide&id=189526639) | CCGCTCACAGCGCCCTCTTC | GGTGCCACATGGGTCCAGGC |
| Nestin | [XM_001919887.2](http://www.ncbi.nlm.nih.gov/entrez/viewer.fcgi?db=nucleotide&id=292622372) | CCCAGACCTGGGGCAGGAGT | TCCTGCCAGGCTTGAGCTGC |
| Nanog | NM_001098392.1 | TCAGCGAGCAGTCGAGAAGGGG | TGACTGGCTCCTCTCCTGCTGTG |

| **Drosophila** | **Gene Identification** | **Fwd Primer** | **Rev Primer** |
| --- | --- | --- | --- |
| VVL | [NM_079224.4](http://www.ncbi.nlm.nih.gov/entrez/viewer.fcgi?db=nucleotide&id=221330914) | CCCTGGGCTCTCGGCTGTCTT | GTTGCTTGACCGATTCGATGCCG |
| Sox N | [NM_079996.2](http://www.ncbi.nlm.nih.gov/entrez/viewer.fcgi?db=nucleotide&id=24582930) | GCCAGTCGCACCTGACCCAC | CCAGGCTGACCTTGCACCCG |
| dmyc | [NM_080323.2](http://www.ncbi.nlm.nih.gov/entrez/viewer.fcgi?db=nucleotide&id=24639495) | TCCGTTTGAGCGGCGGTGAC | GCGGCCACCTCGTCGGTAAG |
| Luna | NM_206089 | CGCCAAGCGGCGGATACACA | GTCAGCTCGTCGCTCCTCGC |
| Vasa | [NM_165103.2](http://www.ncbi.nlm.nih.gov/entrez/viewer.fcgi?db=nucleotide&id=45550980) | CGGCGGTGAACGTCGTGGAA | TTCGACGGCGCCTCGCAAAA |
| Escargot | [NM_057252.3](http://www.ncbi.nlm.nih.gov/entrez/viewer.fcgi?db=nucleotide&id=24584438) | GTGCCCACGCCCACATACCC | CGAGAAGGCCTTGCCGCACA |
| Asense | [NM_057346.4](http://www.ncbi.nlm.nih.gov/entrez/viewer.fcgi?db=nucleotide&id=281359620) | ACACCCGCAATCGCCACCAG | CGCCCCCTGGGCCTCAAAAG |
| Diachate | [NM_079342.2](http://www.ncbi.nlm.nih.gov/entrez/viewer.fcgi?db=nucleotide&id=24664097) | AGCAGCCTGGGCAGCAATGG | ATCCCGGCGAGGAGGTGGAC |
| Miranda | [NM_057943.3](http://www.ncbi.nlm.nih.gov/entrez/viewer.fcgi?db=nucleotide&id=24648310) | GGCGAGGCGGAGACGTTGG | AGAGGCGGATCCCGGTTGCT |
| Snail | [NM_057384.3](http://www.ncbi.nlm.nih.gov/entrez/viewer.fcgi?db=nucleotide&id=24584447) | CCCAGGATCAGCCGCAGGATCTA | CTCGTCGCGACCCCGTTTCA |

**Supplementary file 1D. P-values for the graphs shown in the figures of this study.** All comparisons show Tukey’s post hoc p-values from an ANOVA test, except for titer (Figure 2 – Figure Supplement 2) that shows the overall ANOVA p- value. **Bold**, significantly different at p<0.05; #, approaches significance. iPSC* = Exogenous expression of factors; iPSCs = Endogenous expression of factors; FB = fibroblast, ESC = Embryonic stem cell, EB = Embryoid Body. NA = Not Applicable.

| **Species** | **Gene** | **Figure 3A-F (Passage 2)** | | | **Figure 3G-L (Passage 5)** | | | |
| --- | --- | --- | --- | --- | --- | --- | --- | --- |
|  |  | *iPSC*vs. FB* | *iPSC vs. FB* | *iPSCs vs. ESC* | *iPSC* vs. ESC* | *iPSC vs. FB* | *iPSCs vs. ESC* | *iPSC vs. iPSC** |
| **Mouse** | Oct-4 | **p<0.001** | **p<0.001** | p=0.322 | **p<0.001** | **p<0.001** | p=0.502 | **p<0.001** |
|  | Sox2 | **p<0.001** | **p<0.001** | p=0.299 | **p<0.001** | **p<0.001** | p=0.201 | **p<0.001** |
|  | C-myc | **p<0.001** | **p=0.031** | p=0.520 | **p<0.001** | **p<0.001** | p=0.660 | **p<0.001** |
|  | KLF-4 | **p<0.001** | **p=0.024** | p=0.383 | **p<0.001** | **p<0.001** | p=0.343 | **p<0.001** |
|  | Nanog | NA | **p<0.001** | p=0.320 | **p<0.001** | **p<0.001** | p=0.226 | **p<0.001** |
|  | Vasa | NA | **p<0.001** | p=0.131 | **p<0.001** | **p<0.001** | p=0.404 | **p<0.001** |
|  | | | | |  | | |  |
| **Chicken** | Oct-4 | **p<0.001** | **p<0.001** | p=0.147 | **p<0.001** | **p<0.001** | p=0.564 | **p<0.001** |
|  | Sox2 | **p<0.001** | **p<0.001** | p=0.431 | **p<0.001** | **p<0.001** | p=0.442 | **p<0.001** |
|  | C-myc | **p<0.001** | **p=0.041** | **p<0.001** | **p<0.001** | **p<0.001** | P=0.110 | **p<0.001** |
|  | KLF-4 | **p<0.001** | p=0.339 | p=0.105 | **p=0.028** | p=0.113 | p=0.222 | **p<0.001** |
|  | Nanog | NA | **p<0.001** | **p<0.001** | **p<0.001** | **p<0.001** | **p<0.001** | **p<0.001** |
|  | Vasa | NA | **p<0.001** | p=0.281 | **p<0.001** | **p<0.001** | **p<0.001** | **p<0.001** |
|  | | | | |  | | |  |
| **Quail** | Oct-4 | **p<0.001** | **p<0.001** | NA | NA | **p<0.001** | NA | **p<0.001** |
|  | Sox2 | **p<0.001** | **p<0.001** | NA | NA | **p<0.001** | NA | **p<0.001** |
|  | C-myc | **p<0.001** | p=0.273 | NA | NA | **p<0.001** | NA | P=0.392 |
|  | KLF-4 | **p<0.001** | p=0.101 | NA | NA | p=0.217 | NA | p>=0.512 |
|  | Nanog | NA | **p<0.001** | NA | NA | **p<0.001** | NA | **p<0.001** |
|  | Vasa | NA | **p<0.001** | NA | NA | **p<0.001** | NA | **p<0.001** |
|  | | | | |  | | |  |
| **Z Finch** | Oct-4 | **p<0.001** | **p<0.001** | NA | NA | **p<0.001** | NA | **p<0.001** |
|  | Sox2 | **p<0.001** | **p<0.001** | NA | NA | **p<0.001** | NA | **p<0.001** |
|  | C-myc | **p<0.001** | **p=0.042** | NA | NA | **p<0.001** | NA | p=0.400 |
|  | KLF-4 | **p<0.001** | p=0.238 | NA | NA | p<0.001 | NA | **p<0.001** |
|  | Nanog | NA | **p<0.001** | NA | NA | **p<0.001** | NA | **p<0.001** |
|  | Vasa | NA | **p<0.001** | NA | NA | **p<0.001** | NA | **p<0.001** |
|  | | | | |  | | |  |
| **Zebrafish** | Oct-4 | **p<0.001** | **p=0.015** | NA | NA | **p<0.001** | NA | **p<0.001** |
|  | Sox2 | **p<0.001** | **p<0.001** | NA | NA | **p<0.001** | NA | **p<0.001** |
|  | C-myc | **p<0.001** | **p=0.032** | NA | NA | **p=0.037** | NA | **p<0.001** |
|  | KLF-4 | **p<0.001** | p=0.102 | NA | NA | p=0.679 | NA | **p<0.001** |
|  | Nanog | NA | p=0.412 | NA | NA | p=0.820 | NA | **p<0.001** |
|  | Vasa | NA | **p<0.001** | NA | NA | **p<0.001** | NA | **p<0.001** |

| **Species** | **Gene** | **Figure 3M-N** | |
| --- | --- | --- | --- |
| **Drosophila** |  | *iPSC* vs. S2* | *iPSC vs. S2* |
|  | VVL | **p<0.001** | **p=0.028** |
|  | SoxN | **p<0.001** | p=0.082 |
|  | dMyc | **p<0.001** | **p=0.031** |
|  | Luna | **p<0.001** | p=0.451 |
|  | Asense | NA | p=0.112 |
|  | Dichaete | NA | **p<0.001** |
|  | Escargot | NA | **p=0.029** |
|  | Miranda | NA | p=0.329 |
|  | Snail | NA | **p=0.059^#^** |
|  | Vasa | NA | **p<0.001** |

| **Passage** | **Figure 3O** | |
| --- | --- | --- |
|  | *iPSC vs. FB* | *iPSCs vs. ESC* |
| *Mouse* | | |
| 1 | p=0.238 | **p=0.039** |
| 2 | p=0.120 | p=0.211 |
| 3 | **p<0.001** | p=0.192 |
| 4 | **p<0.001** | p=0.252 |
| 5 | **p<0.001** | p=0.142 |
| *Chicken* | | |
| 1 | p=0.382 | **p<0.001** |
| 2 | p=0.290 | **p<0.001** |
| 3 | **p<0.001** | **p<0.001** |
| 4 | **p<0.001** | p=0.121 |
| 5 | **p<0.001** | p=0.091 |
| *Quail* | | |
| 1 | p=0.263 | NA |
| 2 | p=0.456 | NA |
| 3 | **p=0.021** | NA |
| 4 | **p<0.001** | NA |
| 5 | **p<0.001** | NA |
| *Zebra finch* | | |
| 1 | p=0.123 | NA |
| 2 | p=0.342 | NA |
| 3 | **p<0.001** | NA |
| 4 | **p<0.001** | NA |
| 5 | **p<0.001** | NA |
| *Zebrafish* | | |
| 1 | p=0.331 | NA |
| 2 | p=0.156 | NA |
| 3 | p=0.121 | NA |
| 4 | **p<0.001** | NA |
| 5 | **p<0.001** | NA |

| **Species** | **Figure 3P, Telomerase** | |
| --- | --- | --- |
|  | *iPSC vs. FB* | *iPSCs vs. ESC* |
| **Mouse** | **p<0.001** | p=0.453 |
| **Chicken** | **p<0.001** | p=0.399 |
| **Quail** | **p<0.001** | NA |
| **Zebra Finch** | **p<0.001** | NA |
| **Zebrafish** | **p<0.001** | NA |

| **Species** | **Figure 4B, gene expression** | | |
| --- | --- | --- | --- |
|  | *EB vs. FB* | *EB vs. iPSC* | *FB v. iPSC* |
| **Mouse** | **p<0.001** | **p<0.001** | **p=0.034** |
| **Chicken** | **p<0.001** | **p<0.001** | **p=0.033** |
| **Quail** | **p<0.001** | **p<0.001** | **p=0.042** |
| **Zebra Finch** | **p=0.021** | **p<0.001** | p=0.065 |
| **Zebrafish** | **p=0.029** | **p=0.020** | p=0.059 |

| **Species** | **Supplementary Figure 3** |
| --- | --- |
| **Mouse** | **p<0.001** |
| **Chicken** | **p=0.021** |
| **Quail** | **p=0.034** |
| **Zebra finch** | p=0.078 |
| **Zebrafish** | **p= 0.031** |
|  |  |

**Supplementary file 1E. RNA extraction of adult tissue.** The contents of the table denote the origin of the adult tissues template used to compare the different levels of gene expression versus embryonic fibroblasts and iPSC-like cells.

| Species | RNA |
| --- | --- |
| Mouse | Zyagen Cat MR-201 |
| Chicken | Zyagen Cat CR-201 |
| Quail | made with the SV total RNA isolation kit by promega from adult tissue |
| Zebra Finch | made with the SV total RNA isolation kit by promega from adult tissue |
| Zebra Fish | made with the SV total RNA isolation kit by promega from adult tissue |
